# Blue-Light Stimulation for Myopia Prevention: Only Retinal but Not Optic Disc Stimulation Modulates the Pattern ERG

**DOI:** 10.3390/life15091384

**Published:** 2025-09-01

**Authors:** Isabella Silke Elisabeth Mehler, Sven Pascal Heinrich, Daniel Böhringer, Valentin Simon, Tim Bleul, Sebastian Küchlin, Wolf Alexander Lagrèze, Navid Farassat

**Affiliations:** Eye Center, Medical Center—University of Freiburg, Faculty of Medicine, University of Freiburg, Baden-Württemberg, 79106 Freiburg, Germany; isabella.mehler00@gmail.com (I.S.E.M.); sven.heinrich@uniklinik-freiburg.de (S.P.H.); daniel.boehringer@uniklinik-freiburg.de (D.B.); valentin.simon@uniklinik-freiburg.de (V.S.); tim.bleul@uniklinik-freiburg.de (T.B.); wolf.lagreze@uniklinik-freiburg.de (W.A.L.)

**Keywords:** blue light, myopia prevention, intrinsic photosensitive retinal ganglion cells, pattern electroretinogram, choroidal thickness, axial length

## Abstract

Blue-light stimulation of the optic disc has been suggested as a means of myopia prevention by activating dopaminergic amacrine cells via intrinsically photosensitive retinal ganglion cells. This prospective, adequately powered study investigated this approach by examining its effects on pattern electroretinogram (PERG) N95 amplitude and choroidal thickness (ChT), established biomarkers associated with retinal ganglion cell function and myopia progression, respectively. Forty-six healthy adults received one minute of 450 nm blue-light stimulation to either the optic disc or central retina of the right eye, with the fellow left eye serving as control. PERG responses were measured before and 20 min after stimulation (N = 15 per stimulation location), while ChT, using swept-source optical coherence tomography images, was measured before, 20, and 60 min after stimulation (N = 8 per stimulation location). Only retinal stimulation significantly increased PERG N95 amplitude (baseline 16.16 µV, post-stimulation 17.61 µV [*p* = 0.01]), whereas optic disc stimulation did not (baseline 18.71 µV, post-stimulation 18.81 µV [*p* = 0.76]). Neither optic disc nor retinal stimulation significantly changed ChT at any time point. No significant differences were observed between myopic and non-myopic participants. Our findings do not support the hypothesis that short-duration blue-light stimulation of the optic disc is a viable strategy to activate retinal dopaminergic pathways for myopia prevention.

## 1. Introduction

The global rise in myopia prevalence has become a significant global health concern [1]. A key environmental factor linked to this trend is the amount of time individuals spend outdoors exposed to natural daylight: A substantial body of research has demonstrated a robust inhibitory effect of daylight on both myopia onset and, though more debated, on its progression [2,3]. This has spurred research into the therapeutic potential of light-based interventions, using both the short-wavelength [4] and long-wavelength [5] components that are both abundant in daylight. Blue light is proposed to activate melanopsin-containing intrinsically photosensitive retinal ganglion cells (ipRGCs), which exhibit peak sensitivity around 480 nm [6]. This activation is thought to induce retinal dopaminergic amacrine cells (DACs) to release dopamine [7,8], a neurotransmitter considered crucial for contrast sensitivity, light adaptation, and the inhibition of eye growth [9].

Based on these findings, Amorim-de-Sousa et al. (2021) proposed a novel therapeutic approach involving the stimulation of the optic disc with blue light as a strategy for myopia prevention [10]. They reported that 1 min of blue-light stimulation at a wavelength of 450 nm increased the amplitude of the pattern electroretinogram (PERG) 20 min post-stimulation in young adults. The PERG, particularly the N95 component, is a well-established measure associated with retinal ganglion cell activity, including ipRGCs [11]. In another study, the same group reported that 1 min of blue-light optic disc stimulation significantly increased subfoveal choroidal thickness (ChT) in emmetropes but not myopes 60 min post-stimulation [12]. Choroidal thickness is considered an important biomarker for myopia progression [13] and has been consistently associated with myopia onset, progression, and the efficacy of therapeutic interventions such as atropine and ortho-keratology [14,15]. Specifically, while myopia is linked to thinner choroids, its prevention is associated with choroidal thickening. In a current translational effort, blue-light stimulation of the optic disc is carried out via the screen of a smartphone that is inserted into a virtual reality headset (MyopiaX^®^, Dopavision GmbH, Berlin, Germany).

The need for independent verification or falsification of such findings is paramount, particularly given the recent history of questionable interventions lacking robust evidence or understanding of underlying mechanisms. Recently, the field of myopia prevention has seen an increasing number of clinical trials with a trend towards declining efficacy rates [16,17].

Therefore, the present study aimed to rigorously evaluate the proposed approach of blue-light optic disc stimulation. We investigated the immediate effects of a one-minute exposure to blue light at 450 nm on the N95 amplitude of the PERG and the ChT. To distinguish the specific effects of stimulating the optic disc, we included a positive control condition involving stimulation of the central retina. The rationale for this comparison is based on fundamental neuroanatomy: the PERG N95 originates from retinal ganglion cells [11], whose signal-generating somatodendritic compartments are located within the retina, whereas the optic disc is only composed of their axons. Our primary focus was on the PERG N95 component, as it provides the most direct electrophysiological measure of RGC function related to the central hypothesis.

## 2. Materials and Methods

### 2.1. Study Design and Ethical Approval

This prospective, non-randomized interventional study was conducted between January 2023 and May 2024. All participants provided written informed consent prior to participation. Informed consent was obtained to publish the information/images in an online open access publication. The study has been approved by the Central Ethics Commission in Freiburg (#22-1023). All procedures adhered to the principles of the Declaration of Helsinki.

### 2.2. Participants

Forty-six healthy young adults were recruited. Inclusion criteria were age 16 to 30 years and best-corrected visual acuity of ≤0.0 logMAR. Exclusion criteria included existing ophthalmological, neurological, or psychiatric diseases, corneal astigmatism > 1.0 diopter, current use of dopamine agonists or antagonists, and current or previous participation in any active myopia control treatment.

Participants were assigned to one of two experimental protocols: PERG assessment or ChT/Axial length (AL) assessment. Each protocol involved two stimulation conditions (optic disc or central retina), tested on separate groups of participants. Within each group, participants were further categorized as myopic (average spherical equivalent ≤ −0.5 diopters in both eyes) or non-myopic (average spherical equivalent > −0.5 diopters in both eyes).

**PERG protocol:** Fifteen participants (6 males, 9 females; mean age 25.5 ± 2.3 years; mean visual acuity −0.21 ± 0.08 logMAR; mean AL 23.71 ± 0.59 mm; mean spherical equivalent −0.59 ± 1.09 diopters, 5 myopes (average ≤ −0.5 diopters in both eyes), 10 non-myopes (average > −0.5 diopters in both eyes) were recruited for PERG measurements after blue-light stimulation of the optic disc. Another group of 15 participants (5 males, 10 females; mean age 24.29 ± 3.06 years; mean visual acuity −0.24 ± 0.09 logMAR; mean AL 23.29 ± 0.57 mm; mean spherical equivalent −0.14 ± 0.78 diopters, 5 myopes, 10 non-myopes) were recruited for PERG measurements after blue-light stimulation of an extended part of the central retina (see below). We determined the required sample size using data from Amorim-de-Sousa (2021) [10]. The mean baseline amplitude was 5.2 µV, and the mean amplitude 20 min post-stimulation was 5.9 µV. Furthermore, based on intra-individual SDs observed in routine pattern electroretinogram (PERG) examinations in our clinic, the standard deviation (SD) was estimated to be 14% of the mean. This estimate aligns with previously reported values [18,19,20]. The power and alpha levels were set at 80% and 5%, respectively.

**ChT/AL protocol:** Eight participants (3 males, 5 females; mean age 23.0 ± 3.7 years; mean visual acuity −0.26 ± 0.11 logMAR; mean AL 23.46 ± 0.6 mm; mean spherical equivalent −0.89 ± 1.66 diopters, 4 myopes, 4 non-myopes) were recruited for the measurement of ChT and AL after blue-light stimulation of the optic disc. Another eight participants (2 males, 6 females; mean age 24.5 ± 2.6 years; mean visual acuity −0.22 ± 0.1 logMAR; mean AL 23.66 ± 1.01 mm; mean spherical equivalent −0.95 ± 1.39 diopters, 5 myopes, 3 non-myopes) were recruited for measurements after stimulation of the central retina. Sample size calculation was based on anticipated changes in ChT observed with other myopia prevention strategies, such as atropine, assuming a ChT change of approximately 10 µm, a standard deviation of 5–10 µm, a power of 80%, and alpha of 5%, which indicated a required sample size of n = 8 per group.

### 2.3. General Experimental Procedure

For both the PERG and the ChT/AL protocols, the basic procedure involved a baseline measurement, a blue-light stimulation, and post-stimulation measurements. This was preceded by determination of refraction and assessment of the best-corrected visual acuity of each eye using the Freiburg Visual Acuity and Contrast Test (FrACT). The left eye (OS) served as the unstimulated control (fellow eye) and was occluded with an occlusion patch during stimulation of the right eye (OD, study eye). All electrophysiological and ChT/AL experiments were conducted in a controlled setting with consistent, dim ambient lighting. The participants were instructed not to consume caffeine and nicotine for at least 5 h before the experiments.

### 2.4. Blue-Light Stimulation

**Figure 1:**
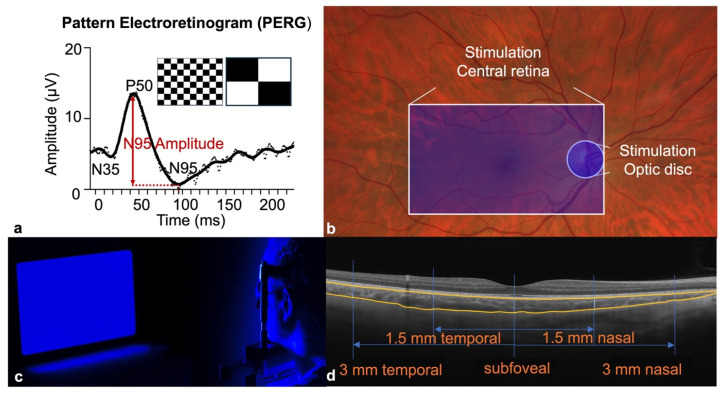
Experimental design: (**a**) Visual stimulus and pattern electroretinogram signal. A transient protocol with check sizes of 0.79° and 17° was used. The N95 amplitude (red) is measured as the difference between the P50 peak and the N95 trough. (**b**) Stimulation area. For stimulation of the optic disc, a blue circle with a radius of 2° was illuminated at the subjectively identified location of the blind spot. For stimulation of the central retina, an area of 18° × 32° was illuminated. (**c**) Blue-light stimulation. Flickering blue-light stimulation (450 nm, 14.7 cd/m^2^, 15 Hz) of the central retina of the right eye in a representative participant. The left eye was covered with an occlusion patch. (**d**) Choroidal thickness measurements. Enhanced depth imaging spectral-domain optical coherence tomography image, demonstrating choroidal thickness measurement locations. Five points were measured: subfoveal, 1.5 mm parafoveal, and 3 mm parafoveal. The measurement start point (lamina basalis choroideae) and end point (lamina suprachoroidea) are outlined in orange.

**Optic disc:** Participants were positioned with their head in a chinrest at 57 cm from an AMOLED display (3840 × 2160 pixels, 15 inch; XtendTouch Pro XT1610U0, Pepper Jobs, Shenzhen, China). They fixated on a small yellow cross at the center of the screen. To locate the blind spot, participants fixated on the central cross while a red target circle (2° radius) was displayed. Participants used the keyboard arrow keys to move the target until it disappeared from their perception, thereby mapping the physiological blind spot corresponding to the optic disc. For stimulation, a blue circle with a radius of 2°, with a wavelength of 450 nm, was presented at a flicker frequency of 15 Hz and brightness of 14.7 cd/m^2^ on average with a duty cycle of 50% on a black background (Figure 1b). The stimulation lasted for 1 min. The full visual stimulation procedure was implemented using PsychoPy3 [21].

**Central retina:** Participants used the same positioning and fixation as in the blind spot stimulation. The entire AMOLED display of the monitor (approximately 18° × 32° on the retina) flickered at 450 nm against a black background with the same luminance and temporal characteristics as with the blind spot stimulation (Figure 1b,c). This large stimulation area was chosen to serve as a robust positive control, maximizing the potential to activate a sufficient number of ipRGCs to elicit a measurable physiological response, thereby validating our experimental setup.

### 2.5. PERG Measurements

The transient PERG was measured at baseline and 20 min after blue-light stimulation in accordance with the International Society for Clinical Electrophysiology of Vision (ISCEV) standard [11] (large-field option). We selected the 20-min post-stimulation time point, as previous studies by Amorim-de-Sousa and colleagues consistently reported the maximal PERG N95 and full-field ERG b-wave amplitudes at this interval [10,22]. A cathode ray tube monitor (1024 × 768 pixels; GD402/21CY9, FIMI-Philips, Saronno, Italy) was used to present the stimulus at a frame rate of 75 Hz and an observation distance of 57 cm. The stimulus consisted of checkerboard patterns with check sizes of either 0.79° or 17° that reversed at a rate of 3.4 per second. Contrast was nearly 100% contrast, and the brightness of the black and white fields was 2.6 cd/m^2^ and 396 cd/m^2^, respectively. The viewing angle covered a 30° field (Figure 1a). Each sweep was 280 ms, the high-pass filter was set to 1 Hz, and the lowpass filter to 100 Hz. The signals were sampled at 1 kHz, and 80 sweeps were averaged. Two measurements were recorded for each condition and averaged for analysis. These experiments were conducted between 10 AM and 5 PM in order to minimize the effects of diurnal variations on dopamine levels.

### 2.6. Choroidal Thickness and Axial Length Measurements

Measurements of ChT and AL were performed at baseline and at 20 min and 60 min after the stimulation. These measurements were conducted between 6 PM and 9 PM to minimize the effect of large diurnal fluctuations in ChT and retinal dopamine.

Choroidal thickness was measured using swept-source optical coherence tomography (Zeiss PlexElite 9000, Carl Zeiss Meditec, Jena, Germany). The raster line scan and HD Spotlight 1 mode with 100 kHz frequency, 100× averaging, and EDI (Enhanced Depth Imaging) mode were used. The scan length was 9 mm. ChT was defined as the distance between the lamina basilaris and the lamina suprachoroidea (Figure 1d). Measurements were obtained subfoveally, and at 1.5 mm and 3 mm parafoveally, both temporally and nasally. Three independent, blinded examiners performed the measurements after anonymization and mirroring of left eye data. Both eyes were measured, with the OD serving as study eye and OS as fellow eye. AL was measured with the IOL Master 700 (Carl Zeiss Meditec, Jena, Germany).

### 2.7. Statistical Analysis

Statistical analysis was performed using GraphPad Prism 10 (GraphPad Software, San Diego, CA, USA). No data points were removed as outliers. The Shapiro–Wilk and Kolmogorov–Smirnov tests revealed that the PERG measurements, as well as ChT and AL measurements, did not follow a normal distribution. Therefore, the Wilcoxon signed-rank test for paired (within subjects) data was used to compare baseline measurements to those taken 20 and 60 min after stimulation. Where appropriate, effect sizes were calculated using Cohen’s d. No adjustments were made for multiple comparisons. To assess the reliability of the ChT measurements, the intraclass correlation coefficient (ICC) was calculated using a two-way consistency model across the three examiners. Unless otherwise stated, all data are given as mean ± SD.

## 3. Results

We enrolled a total of 46 healthy volunteers between January 2023 and May 2024, 30 of whom were assigned to the PERG protocol, and 16 of whom followed the ChT and AL protocol. Their characteristics are shown in Table 1.

### 3.1. PERG Response After Blue-Light Stimulation of the Optic Disc

**Figure 2:**
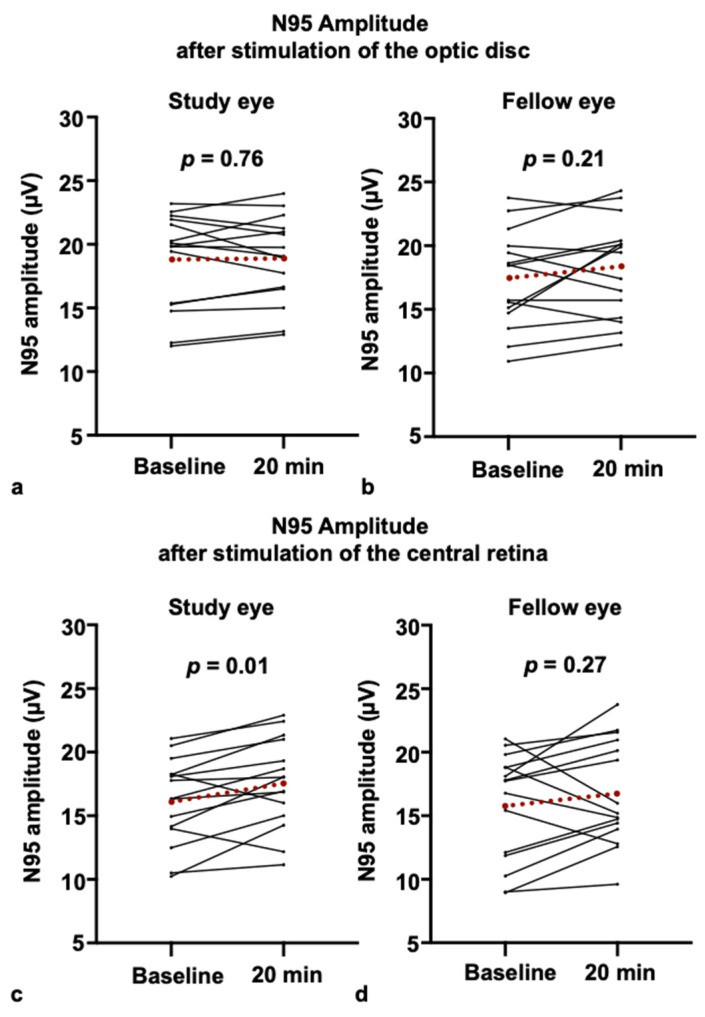
Only central retinal but not optic disc stimulation increases N95 amplitude of the pattern electroretinogram: (**a**,**b**) N95 amplitudes of the study eye (**a**) and fellow eye (**b**) after optic disc stimulation. (**c**,**d**) N95 amplitudes of the study eye (**c**) and fellow eye (**d**) after central retinal stimulation. Values for individual participants are shown in black. The mean values are shown in red.

Our primary analysis sought to determine if blue-light stimulation of the optic disc could replicate previously reported increases in RGC activity. For the N95 amplitude at a check size of 0.79°, after one minute of blue-light stimulation of the optic disc OD, there was no statistically significant difference between the baseline measurement and the measurement 20 min post-stimulation in either eye. In the study eye, the mean amplitude changed from 18.71 (±3.77) µV at baseline to 18.81 (±3.43) µV post-stimulation (*p* = 0.76), representing a negligible effect size (Cohen’s d = 0.03). Similarly, the unstimulated fellow eye showed no significant change (baseline 17.37 (±3.81) µV, 20 min post-stimulation 18.28 (±3.87) µV [*p* = 0.21]) (Figure 2a,b). Furthermore, we observed no statistically significant differences between baseline and post-stimulation measurements for the N95 amplitude at a check size of 17°, and the N95 peak times at 0.79° and 17°. Given that prior research [10] reported an effect primarily in myopic individuals, we performed an exploratory subgroup analysis. In our myopic cohort (n = 5), the mean N95 amplitude in the study eye changed from 19.15 (±3.73) µV at baseline to 18.35 (±3.0) µV post-stimulation (*p* = 0.125), a non-significant trend in the opposite direction of the previously reported effect. Our non-myopic cohort (n = 10) also showed no significant change (baseline 18.49 ± 3.58 µV, post-stimulation 19.03 ± 3.44 µV; *p* = 0.16).

### 3.2. PERG Responses After Blue-Light Stimulation of the Retina

In contrast to optic disc stimulation, our positive control condition involving wide-field retinal stimulation did elicit a change in RGC activity: Following a one-minute blue-light stimulation of the retina, we observed a statistically significant increase in the N95 amplitude to 0.79° checks for the study eye (baseline 16.16 (±3.38) µV, 20 min post-stimulation 17.61 (±3.53) µV [*p* = 0.01]), with a moderate effect size (Cohen’s d = 0.42). There was no statistically significant change for the fellow eye (baseline 15.81 (±4.24) µV, 20 min post-stimulation 16.78 (±4.15) µV [*p* = 0.27]) (Figure 2c,d). There were no differences between myopic and non-myopic participants.

### 3.3. Choroidal Thickness and Axial Length After Blue-Light Stimulation

**Figure 3:**
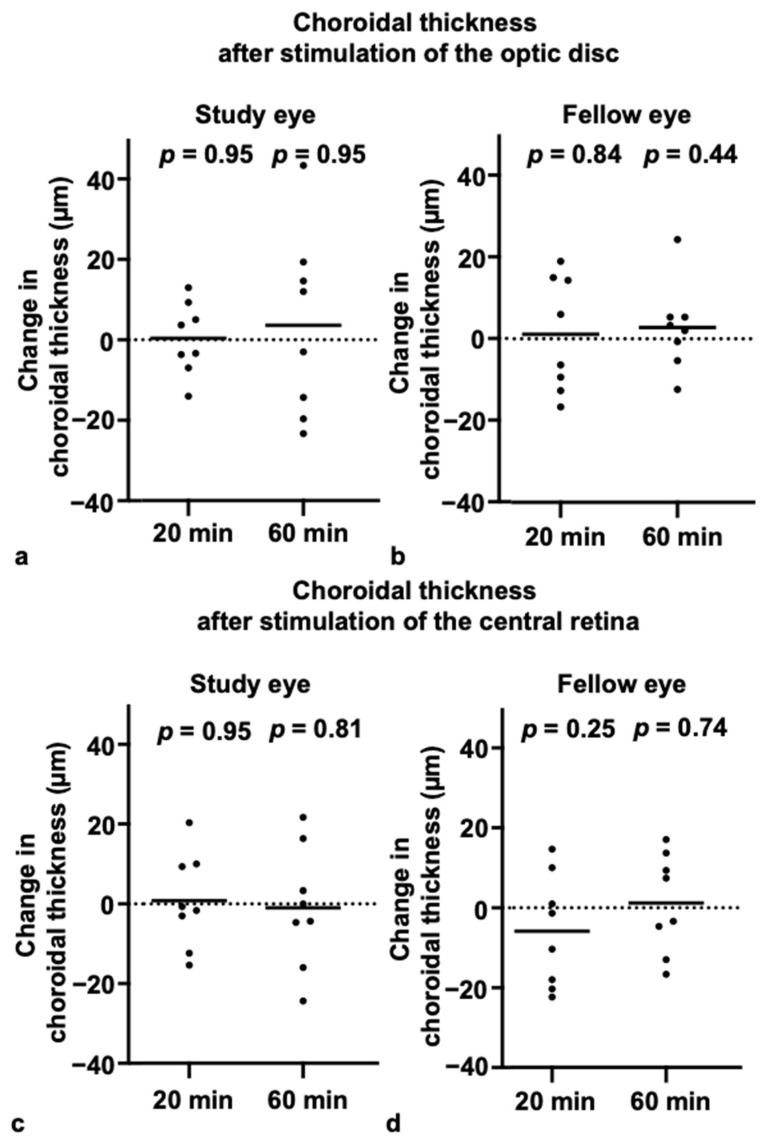
No change in choroidal thickness after blue-light stimulation of the optic disc and central retina: (**a**,**b**) Changes in choroidal thickness of the study eye (**a**) and fellow eye (**b**) at 20 and 60 min after optic disc stimulation. (**c**,**d**) Changes in choroidal thickness of the study (**c**) and fellow eye (**d**) at 20 and 60 min after central retinal stimulation. Lines represent the mean.

**Figure 4:**
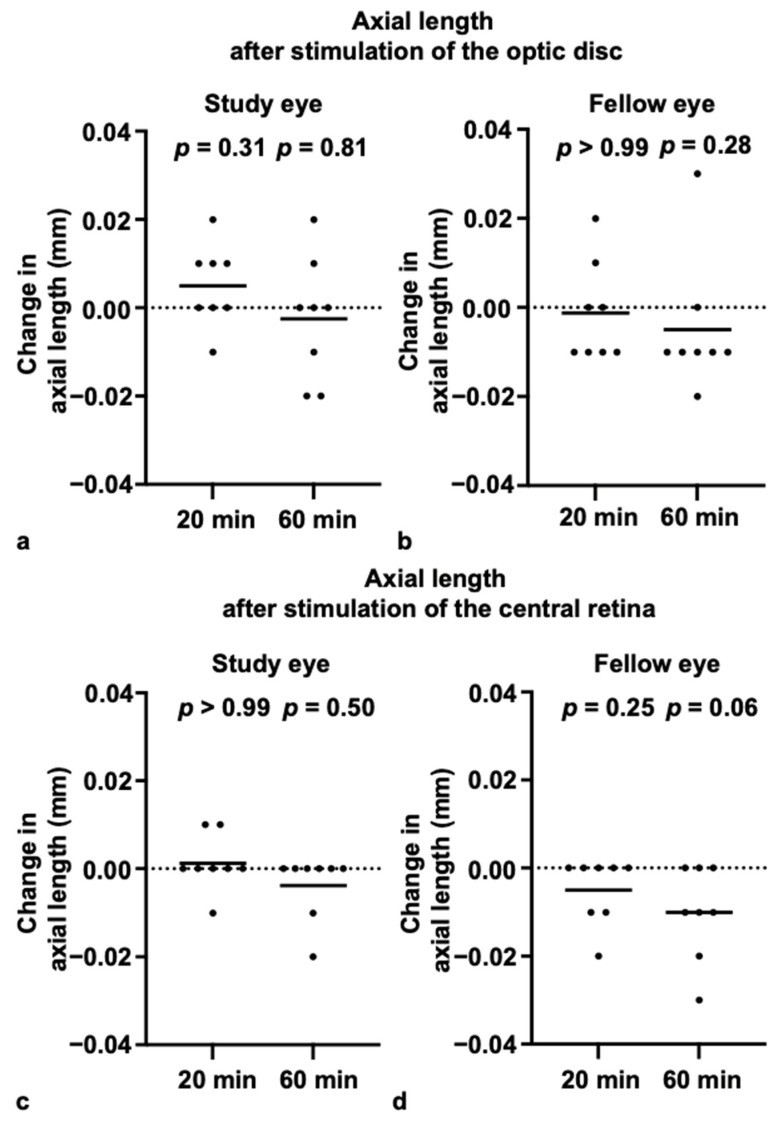
No change in axial length after stimulation of the optic disc and central retina: (**a**,**b**) Changes in axial length of the study (**a**) and fellow eye (**b**) at 20 and 60 min after optic disc stimulation. (**c**,**d**) Changes in axial length of the study (**c**) and fellow eye (**d**) at 20 and 60 min after central retinal stimulation. Lines represent the mean.

There were no statistically significant differences in either subfoveal ChT or AL compared to baseline, at either 20 or 60 min after blue-light stimulation of either the optic disc or the retina (Figure 3 and Figure 4) (ChT—optic disc stimulation: study eye change from baseline: 20 min, 0.38 (±8.97) µm [*p* = 0.95]; 60 min, 3.63 (±22.83) µm [*p* = 0.95]; fellow eye change from baseline: 20 min, 1.17 (±14.1) µm [*p* = 0.84]; 60 min, 2.75 (±10.59) µm [*p* = 0.44]; ChT—retinal stimulation: study eye change from baseline: 20 min, 0.83 (±11.92) µm [*p* = 0.95]; 60 min, −1 (±15.25) µm [*p* = 0.81]; fellow eye change from baseline: 20 min, −5.83 (±14.1) µm [*p* = 0.25]; 60 min, 1.21 (±12.45) µm [*p* = 0.74]; AL—optic disc stimulation: study eye change from baseline: 20 min, 0.005 (±0.01) mm [*p* = 0.31]; 60 min, −0.003 (±0.01) mm [*p* = 0.81]; fellow eye change from baseline: 20 min, −0.001 (±0.01) mm [*p* > 0.99]; 60 min, 0.005 (±0.02) mm [*p* = 0.28]; AL—retinal Stimulation: study eye change from baseline: 20 min, 0.001 (±0.01) mm [*p* > 0.99]; 60 min, −0.004 (±0.01) mm [*p* = 0.50]; fellow eye change from baseline: 20 min, −0.005 (±0.01) mm [*p* = 0.25]; 60 min, 0.01 (±0.01) mm [*p* = 0.06].

Consistent with the subfoveal findings, no statistically significant changes from baseline were observed at any of the parafoveal locations (at 1.5 mm and 3 mm, both temporal and nasal to the fovea), in either the study or the fellow eye, at either the 20- or 60-min time point (all *p* > 0.05). Also, there were no differences between myopic and non-myopic participants. The intraclass correlation coefficient (ICC) for choroidal thickness measurements among the three examiners indicated very good reliability (0.963).

## 4. Discussion

This study investigated the immediate effects of blue-light stimulation, delivered to either the central retina or the optic disc, on the PERG, ChT, and AL. We found a modest but statistically significant increase in the PERG N95 amplitude 20 min after retinal stimulation, while optic disc stimulation failed to elicit any significant response. Furthermore, neither stimulation paradigm resulted in significant changes in ChT or AL. These results were consistent across both myopic and non-myopic participants.

Our findings regarding optic disc stimulation contrast with those reported by Amorim-de-Sousa et al. (2021) [10], who reported an increase in N95 amplitude 20 min after similar blue-light stimulation of the optic disc. Our study was prospectively powered to detect an effect of the size reported by Amorim-de-Sousa et al. (2021) [10]. The failure to find a significant effect (*p* = 0.76), combined with a negligible observed effect size (d = 0.03), strongly suggests that a 1-min blue-light stimulation of the optic disc does not produce an acute PERG N95 increase of the previously reported magnitude. Notably, this increase in N95 amplitude in their paper was specific to myopic subjects and, to our knowledge, has not been independently replicated. While we adhered as closely as possible to their published protocol (with regard to stimulation wavelength (450 nm in both protocols), duration (1 min in both protocols), luminance (14.7~15 cd/m^2^ in our/their protocol) and flicker frequency (15 Hz in both protocols) as well as post-stimulation intervals (20 min)), some methodological differences exist that must be considered. Specifically, we stimulated only one eye, using the fellow eye as an internal control, while Amorim-de-Sousa et al. stimulated both eyes simultaneously [10]. We also used a standard AMOLED monitor for light stimulation instead of a VR headset. While these subtle variations could contribute to the discrepancy, the absence of independent replication of the original findings raises concerns about the robustness of the initial report.

The differences in PERG responses we observed following retinal versus optic disc stimulation likely reflect the distinct cellular compositions of these regions. The retina comprises a complex network of photoreceptors and neurons, including ipRGCs, whose cell bodies are directly exposed to light. Conversely, the optic disc primarily consists of retinal ganglion cell axons, including those of ipRGCs, and is devoid of rods and cones. While few anatomical investigations in non-human animals suggest that ipRGC axons at the optic disc may express melanopsin [4,23], direct evidence for functionally significant levels of melanopsin expression at the human optic nerve head is currently lacking, and the capacity for retrograde axonal activation to modulate somatic RGC activity remains speculative. Our hypothesis is therefore that the density of melanopsin-expressing structures and/or the efficiency of any potential retrograde signaling from the optic disc is insufficient to induce a substantial change in the activity of the retinal dopaminergic system and the PERG response. This is of particular importance given the hypothesis that blue-light optic disc stimulation could be a viable approach for myopia management, an idea explored in several publications by Amorim-de-Sousa and colleagues [4,12,22,24,25]. These works suggest that blue-light stimulation of the optic disc can activate melanopsin, improve contrast sensitivity, increase retinal activity, and increase choroidal thickness, with potentially beneficial effects on myopia progression. These claims are supported by findings of pupillary responses, changes in contrast sensitivity, and ERG and PERG changes, as well as short-term increases in choroidal thickness after blue-light optic disc stimulation in human participants. Furthermore, a digital application (MyopiaX^®^, Dopavision GmbH, Berlin, Germany) has been developed, using a smartphone mounted in a VR headset, to provide this type of stimulation, and a clinical trial is currently being conducted to test its efficacy in myopic children (NCT04967287).

It is crucial to critically evaluate the underlying premise of this approach. The evidence for substantial melanopsin expression specifically at the optic disc in humans is currently limited. Moreover, even if melanopsin is present, the question remains whether short-duration stimulation at this location alone can robustly activate DACs and induce clinically relevant effects.

Our positive findings in the control condition of retinal as opposed to optic disc stimulation align well with existing evidence that DACs are modulated by multiple retinal inputs. Excitatory signals originate from ipRGCs and ON pathway cones through glutamate, while rods and OFF pathway cones exert inhibitory effects through glycine and GABA, respectively [26]. The 1.45 µV increase in PERG amplitude we observed after retinal stimulation, while modest, serves as an important positive control. It confirms that our experimental setup could detect a physiological response to blue light, thereby strengthening our negative finding for optic disc stimulation. The purpose of this short-term experiment was not to elicit a large, clinically meaningful effect, but rather to test the underlying hypothesis of whether the stimulation protocol could engage the target retinal pathways at all. The approach of Amorim-de-Sousa et al. (2021) argues that isolated stimulation of the optic disc avoids inhibitory effects on dopamine release [10]. However, the PERG is influenced by both the somatodendritic and axonal compartments of RGCs [27]. This may explain why only retinal stimulation induced a measurable PERG change in our study, while optic disc stimulation failed to produce a comparable effect.

Importantly, we found no significant changes in either choroidal thickness or axial length after either retinal or optic disc stimulation. The choroid, considerably modulated by dopamine [28], is a widely recognized biomarker for myopia progression [29,30,31]. The suggested link between choroidal thickening, reduced scleral hypoxia, and the suppression of axial elongation [5] emphasizes the potential importance of ChT changes for myopia control. The lack of changes in our study contrasts with other research that showed changes in choroidal thickness with wavelength-specific light exposure [32,33]. For instance, Thakur et al. (2021) demonstrated that blue light was able to counter the effects of hyperopic defocus on axial length in human adults, albeit after one hour of illumination of the posterior segment not limited to the optic disc [33]. In addition, evidence from animal models supports the idea that blue light can induce an increase in choroidal thickness [34].

Red light, on the other hand, has also been implicated in choroidal thickening and myopia prevention in some human studies [35,36,37]. These findings highlight the need for a nuanced view of how different wavelengths of light and the site of stimulation can impact choroidal thickness in distinct species.

Several limitations of our study warrant consideration. First, our methodology differed from that of Amorim-de-Sousa et al. (2021) regarding the use of a VR headset and unilateral stimulation, as discussed above [10]. Second, our study employed a non-randomized design, which could introduce potential selection bias. Third, we did not apply a statistical correction for multiple comparisons, such as for the multiple ChT measurements. Our study was powered for a single, pre-specified primary hypothesis regarding the PERG response. To avoid inflating the risk of a Type II error for our secondary, exploratory analyses, we opted against a strict correction. The fact that no significant changes were found even with this approach strengthens our null findings. Fourth, our interpretation must be carefully framed in the context of the subgroup-specific findings of Amorim-de-Sousa et al. [10], who reported an effect primarily in myopic participants. Our power calculation was based on the large effect size observed in their myopic cohort, and our study was adequately powered to detect such an effect in our overall group. While our study was not prospectively powered to definitively confirm or falsify an effect within the myopic subgroup alone, our exploratory analysis of this group is informative: We found that, contrary to the original report, our myopic participants showed no increase in N95 amplitude; in fact, the data showed a non-significant trend in the opposite direction. This finding, combined with the complete absence of a signal in our overall group and a negligible effect size (d = 0.03), casts significant doubt on the robustness and reproducibility of the originally reported effect. Fifth, our PERG measurements were limited to two time points (baseline and 20 min post-stimulation), as previous studies have reported the greatest response at this time [4,22]. Similarly, our measurements of ChT and AL were restricted to three time points and conducted on a small group of participants. Although our sample size was based on an a priori power calculation to detect clinically relevant changes of ~10 µm in ChT, our study may have been underpowered to detect more subtle changes in ChT and especially AL, and our negative findings for these outcomes should be interpreted with caution.

In addition, our stimulation duration was only one minute, and measurements were limited to baseline and distinct time points post-stimulation. Therefore, our results cannot be used to draw conclusions about the potential efficacy of longer or repeated stimulation sessions.

Finally, myopia progression is a chronic process, and any effective preventative strategy would likely require sustained or cumulative effects over months or years to produce clinically meaningful changes in axial elongation and refractive error. Due to this reason, our focus on short-term responses does not allow direct conclusions to be drawn about long-term treatment effects and whether this approach can induce beneficial effects on axial length and refractive error.

In conclusion, while blue-light stimulation of the central retina induced a modest increase in the PERG response, our findings do not provide support for the hypothesis that short-duration blue-light stimulation of the optic disc is a viable approach for acutely modulating ipRGC activity or choroidal thickness for myopia prevention. The small sample sizes, particularly for the choroidal thickness measurements, limit the statistical power and call for caution when drawing definitive conclusions.

Given the discrepancies with prior reports and the limitations of short-term measurements, a cautious interpretation of the existing literature is warranted. Further research, potentially employing different stimulation parameters (e.g., longer duration or repeated exposures) and long-term clinical endpoints, is necessary to fully evaluate the therapeutic potential of this novel approach for myopia prevention.

## Figures and Tables

**Table 1:** Characteristics of participants: Values are presented as mean and standard deviation (SD) for continuous variables (e.g., age, visual acuity, axial length) and as count (N) and percentage (%) for categorical data (e.g., sex).

Characteristics	General Study Population		PERG Protocol Only		ChT/AL Protocol Only	
	n = 46		n = 30		n = 16	
	Mean/N	SD/(%)	Mean/N	SD/(%)	Mean/N	SD/(%)
**Age** (years)	24.25	2.90	24.75	2.66	23.75	3.13
**Sex** Female Male						
	16	34.78	19	63.33	11	68.75
	30	65.22	11	36.67	5	31.25
**Visual acuity** (logMAR)	−0.23	0.10	−0.23	0.09	−0.24	0.11
**Axial length** (mm)	23.53	0.69	23.50	0.58	23.56	0.81
**Spherical equivalent** (diopters)	−0.64	1.23	−0.37	0.94	−0.92	1.53
**Refraction** Myopes Non-Myopes						
	19	41.30	10	33.33	9	56.25
	27	58.70	20	66.67	7	43.75

## Data Availability

The data presented in this study are available on request from the corresponding author. The data are not publicly available due to privacy and ethical restrictions. Anonymized data may be made available by the corresponding author upon reasonable request.

## Abbreviations

The following abbreviations are used in this manuscript:

ipRGC	Intrinsically photosensitive retinal ganglion cells
DAC	Dopaminergic amacrine cells
PERG	Pattern electroretinogram
ChT	Choroidal thickness
AL	Axial length
OS	Left eye
OD	Right eye
FrACT	Freiburg Visual Acuity and Contrast Test
ICC	Intraclass correlation coefficient
VR	Virtual reality
